# The Use of Inertial Measurement Units for the Study of Free Living Environment Activity Assessment: A Literature Review

**DOI:** 10.3390/s20195625

**Published:** 2020-10-01

**Authors:** Sylvain Jung, Mona Michaud, Laurent Oudre, Eric Dorveaux, Louis Gorintin, Nicolas Vayatis, Damien Ricard

**Affiliations:** 1 Université Paris-Saclay, ENS Paris-Saclay, CNRS, Centre Borelli, F-91190 Gif-sur-Yvette, France; sylvain.jung@engie.com (S.J.); michaudmona@gmail.com (M.M.); nicolas.vayatis@cmla.ens-cachan.fr (N.V.); damien.ricard@m4x.org (D.R.); 2 Université de Paris, CNRS, Centre Borelli, F-75005 Paris, France; 3 Université Sorbonne Paris Nord, L2TI, UR 3043, F-93430 Villetaneuse, France; 4 ENGIE Lab CRIGEN, F-93249 Stains, France; eric.dorveaux@engie.com (E.D.); louis.gorintin@engie.com (L.G.); 5 Service de Neurologie, Service de Santé des Armées, Hôpital d’Instruction des Armées Percy, F-92190 Clamart, France; 6 Ecole du Val-de-Grâce, Ecole de Santé des Armées, F-75005 Paris, France

**Keywords:** wearable sensors, IMUs, accelerometers, gyroscopes, physical activity, free-living environment

## Abstract

This article presents an overview of fifty-eight articles dedicated to the evaluation of physical activity in free-living conditions using wearable motion sensors. This review provides a comprehensive summary of the technical aspects linked to sensors (types, number, body positions, and technical characteristics) as well as a deep discussion on the protocols implemented in free-living conditions (environment, duration, instructions, activities, and annotation). Finally, it presents a description and a comparison of the main algorithms and processing tools used for assessing physical activity from raw signals.

## 1. Introduction

Noncommunicable Diseases (NCDs), also known as chronic diseases, tend to be of long duration and result from combined physiological, genetic, behaviors, and environmental factors. This group of diseases, consisting of several conditions such as cardiovascular disorders, diabetes, certain types of cancers, and chronic respiratory illnesses, kills 41 million people each year, equivalent to 71% of all deaths globally [1]. Some behaviors such as physical inactivity, unhealthy diet, or overweight increase the risk of NCDs. Insufficient Physical Activity (PA) itself is believed to cause 1.6 million deaths annually, hence the growing need to monitor and to assess more closely physical behaviors. Energy Expenditure (EE) estimation is often used as a way to assess the PA of a subject. To achieve this level of detail and compute as precisely as possible, the EE activity classification enables the detection of the exact types of activity that a subject is performing and associates it with an EE value [2].

Falls are also a major health issue to elderly people resulting often in hip fracture requiring surgical operation and long rehabilitation. One-third of people over the age of 65 fall at least once a year. In 2007, half of those over 80 fell at least once a year [3]. Among seniors, falls are a recurring problem in clinical practice as they involve a loss of independence and mobility on top of causing fractures and serious injuries. It is suggested that neuromuscular coordination, muscle strength steadiness of gait, postural stability, and the structural properties of bone all influence fall frequency [4]. This highlights once again the importance of being able to assess the level of PA of certain populations, particularly in environments where their daily activities take place. Some studies are particularly oriented towards the study of cohorts affected by pathologies impacting this PA such as neurological diseases (Parkinson’s Disease (PD) [5] and multiple sclerosis), Chronic Obstructive Pulmonary Disease (COPD) [6,7], or traumas such as strokes.

PA was traditionally assessed by questionnaires [8], but the limitations of this qualitative approach needed to be addressed and notably through the use of more objective monitors. Portable inertial sensors such as Inertial Measurement Units (IMUs), accelerometers, and gyroscopes are currently one of the most widely used solutions for assessing the physical condition of a subject. These small and inexpensive sensors enable an optimized assessment of gait characteristics [9,10,11,12]. They can be used individually or combined to provide complementary information. In this article, we distinguish three main types of sensors: accelerometers, gyroscopes, and IMUs. An accelerometer is a sensor measuring the linear acceleration in one or several directions of space at a given point. These sensors can be used to estimate the static position of the body [13] as well as to detect [14] and study certain movements, particularly in open environments. Gyroscopes measure the angular velocities in one or more directions of space at a given point [5,15,16]. The latter can be used to compute specific features such as angles between different parts of the body [17,18] or Range of Motion (ROM) of the trunk to assess the stability of a subject’s gait [19]. Finally, IMUs are portable systems integrating accelerometers, gyroscopes, and magnetometers that allow the synchronized measurement of linear accelerations and angular velocities in one single device. Moreover, in IMUs, thanks to the magnetometer that measures the absolute magnetic field, all relative quantities (such as accelerations and angular velocities) can be projected into an absolute analysis frame, which might convenient in several situations. All these sensors may be 1D (thus measuring the quantities of interest in only one direction), but most of recent studies rely on 3D sensors, in which the accelerations, angular velocities, and magnetic fields are recorded in the three dimension of space.

The first uses of such sensors for physical observations were from the 1970s and the 1980s [20,21,22,23]. One conclusion from the “Measurement of Physical Activity” meeting conducted by the Cooper Institute in 1999 was that the objective quantification from such sensors was not practical for large-scale, was expensive, and that the acquired data was hard to manipulate or interpret [24]. Since then, progress has increased the cost-effectiveness of these sensors as well as their ease of implementation. These different types of sensors have since been widely used for PA estimation and gait analysis for more than 30 years. The first works with such sensors were conducted in controlled settings [22] and most of the following similar works were carried out under the same conditions. Several tasks were performed in these laboratory settings such as activity classification [25], gait analysis [12,26], or EE estimation [27,28]. One of the shortcomings of this type of environment is that it can cause a Hawthorne effect to appear, which affects the performance of the participants as they are aware throughout the measurements that their PA is being analyzed. Conducting the same analysis in freer environments (or even in environments that are more usual for the subjects) can help to mitigate the impact of this effect [29,30]. The first studies to be referenced as free-living studies using motion sensors were assessing PA levels of specific cohorts [31,32]. Since these pioneer works, numerous studies have used inertial sensors for the assessment of PA in free-living settings, introducing a wide spectrum of experimental conditions. Those works which are performed in free-living settings had to face various issues and challenges [33,34]. In 1999, a National Institute of Health Expert Panel raised some concerns about the inability of studies performed at this period of time or prior to it to produce reliable evaluations of PA because of, among other things, the difficulties in measuring PA in free-living settings [35]. It has also been shown that motion sensors such as accelerometers could underestimate EE compared to other existing methods at this time [36]. In addition, some studies have highlighted the difficulties of transposing high-performance algorithms intended for controlled environments to free-living conditions, in particular because of the greater complexity of movements and their associations [33]. Nevertheless, these challenges have been, and are still being, met today through studies allowing interesting and reliable quantitative feedback using inertial sensors of the movements carried out in free-living settings [37,38,39,40]. The increasing use of machine learning and efficient algorithms also contributed to this effort, by allowing the processing of complex data and allowing to transpose protocols from laboratory to natural conditions. Indeed, although some studies are focused on Semi-Free-Living Environments (semi-FLEs) [15,41,42,43] where the conditions are controlled by the experimenter, more and more research works are being dedicated to Free-Living Environments (FLEs) which use the subjects’ natural environment [5,6,44].

In this context, the goal of our review is to identify and describe the uses of portable inertial sensors in FLEs or semi-FLEs. It will also specifically include research work that includes phases of movement, particularly walking. It aims at providing a selective yet complete overview on the topic, by reviewing technical aspects linked to the used sensors, behavioral aspects such as protocols and instructions and mathematical aspects with a description of the main features and algorithms used for PA estimation. To that end, we propose to look back on the ten most recent years of research on these themes from the early pioneer works to the recent deep learning approaches.

### 1.1. Existing Reviews

Several reviews have endeavored to identify the various studies using motion sensors to analyze the PA of subjects, taking into account at least partially if not exclusively works carried out under free-living conditions. Gorman et al. [45] have detailed several methods of EE assessment in free-living settings. De Bruin et al. [46], Byrom and Rowe [47] (COPD patients), Tedesco et al. [48], Murphy [49], de Oliveira Gondim et al. [50], and Frechette et al. [51] considered the use of wearable systems (accelerometers or other motion sensors) to monitor activities in specifically targeted cohorts (PD, Multiple Sclerosis (MS)). Vienne et al. [19], Yang and Hsu [52], and Tedesco et al. [48] also focused on the use of wearable sensors in clinical settings, but considered any kind of cohort. Attal et al. [53] and Narayanan et al. [54] analyzed articles related to activity classification algorithms and classifiers. Schwickert et al. [55] and Henriksen et al. [56] focused on other kinds of studies (studies based on fall detection and studies using one specific brand of sensors, respectively).

Table 1 summarizes all reviews that have addressed some of the pivotal topics of this paper (motion sensors, free-living settings, etc.). It specifies whether the referenced review articles have addressed the three main aspects detailed in this review: sensors, protocols, and algorithms. It is noteworthy that, according to Table 1, the majority of the reviews selected for this section generally includes articles explaining the various aspects of sensor implementation in a detailed manner, whereas they tend to be less detailed regarding the implementation of protocols (instructions, measurement durations, etc.).

### 1.2. Scope and Limitations of This Review

This review aims to give a complete overview of studies concerning PA assessment using inertial motion sensors in FLEs or semi-FLEs. Besides, this article intends to focus on assessments that include motion phases from the lower limbs such as walking, although there are very few studies that do not include such movement phases. In addition, a section entirely devoted to protocols was put in place in order to detail what is currently conducted in the field.

This review only includes articles related the assessment of PA of participants in free-living settings and will not address locomotive tests classically used in controlled conditions (such as the 6 MWT, 10 m test, etc.) or any other test that can be only performed in controlled settings. Although a significant part of the articles concern pathologies, an in-depth study of the differences between pathologies will not be carried out. In addition, we endeavor to detail the acquisition modes employing a specific type of sensor: inertial motion sensors (gyroscopes, accelerometers, and IMUs). Articles focusing mainly on other types of sensors such as GPS, pressure sensors, or heart rate sensors are not included, which therefore constitutes a limitation to our study. In addition, this review does not perform a complete analysis of all research detailing activity classification processes. Indeed, the willingness to focus on papers that include the use of inertial sensors as well as papers based on open environments does not allow for such a thorough review. Besides, no conditions other than free-living conditions are under consideration in this study, which again deliberately limits the scope of this review. Finally, it is to be noted that Google Scholar articles whose duplicates were not found in the PubMed library were not included in this review (further details are provided in Section 1.3).

### 1.3. Methodology

This scoping selective review has been conducted by searching MEDLINE via PubMed and Cochrane electronic databases to identify articles published from 1 January 2010 to 21 April 2020 whose methods were including the use of wearable sensors such as IMUs, accelerometers, or gyroscopes in order to perform an analysis on participants gait and PA in FLEs. Articles that were not directly exploiting output data from these sensors, or that were not performing their protocols in FLEs or at least in semi-FLEs were excluded from the review. According to the scope detailed in Section 1.2, the following terms were looked after in titles, keywords, abstracts: (((‘’IMU” or ‘’accelerometer” or ‘’gyroscope” or ‘’inertial sensor”) and (‘’free-living” or ‘’outpatient” or ‘’real life” or ‘’out-of-laboratory”)) and (‘’walk” or ‘’gait”).

**Figure 1:**
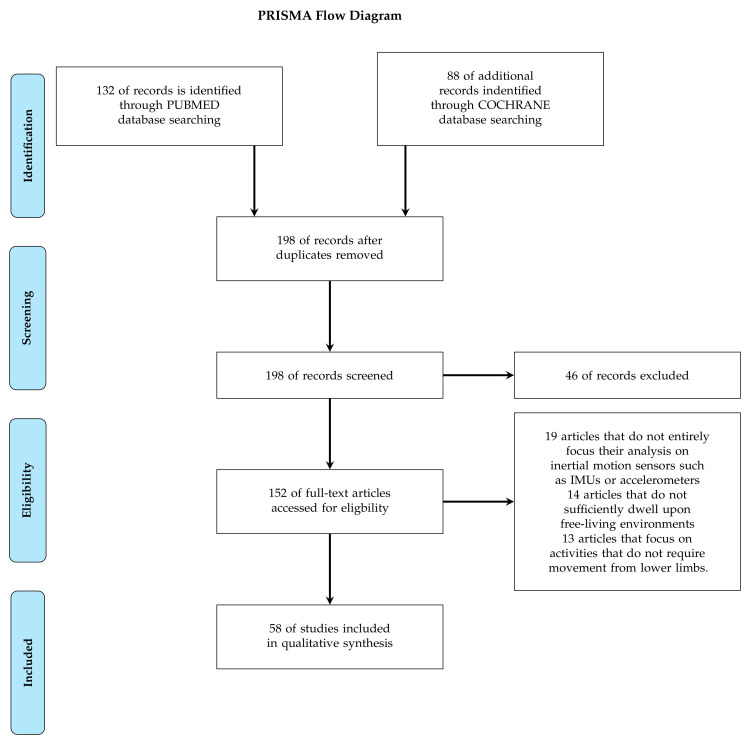
PRISMA flow chart illustrating the selection process resulting in a list of 58 studies.

This selective review was conducted by using Preferred Reporting Items for Systematic Review and Meta-Analyses PRISMA guidelines to select articles as detailed in Figure 1. Potentially eligible studies were screened individually by three authors (MM, SJ, and LO) on the basis of abstract and title for FLEs and wearable sensors criterion and of the full text for others criterion. In total, 58 articles meeting the search criteria detailed above were finally selected for this review. This work had first been focusing on the Google Scholar library before it was noticed that the vast majority of the articles that were being selected were duplicated in the PUBMED library (most of studies based on activity classification had clinical objectives: activity observation, physical activity evaluation, etc.). This is why it has been decided to only mention the Pubmed library in the PRISMA Flow Chart since google scholar library is not specifically specified on this PRISMA model in other studies from the literature [58,59].

### 1.4. Organization of the Paper

The paper is organized as follows. Section 2 provides a first rough classification of the 58 selected articles by describing the reported aims of the studies. Section 3 provides an investigation on the sensors, by detailing the type, number, and placement used in the studies, as well as some technical considerations. Section 4 gives a thorough description of the protocols used in FLEs including instructions, activities, inclusion criteria, etc. Section 5 is related to the algorithms and features used for activity classification and PA assessment. Finally, Section 6 provides a discussion on the current challenges and open questions in this research field.

### 1.5. Results of Screening

As a conclusion, 58 articles were kept for a further analysis. The selection process put in place in regards to the PRISMA flowchart is detailed in Figure 1.

## 2. Aims of the Studies

**Figure 2:**
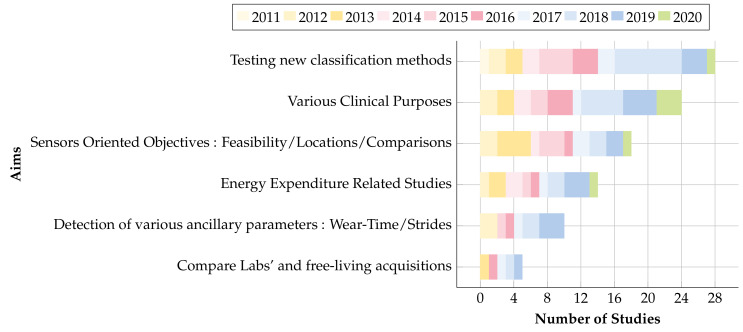
Change of the distribution of studies according to their aims over time.

In this review, 58 studies linked to the use of IMUs in the context of FLEs were included. However, these studies have various objectives, which range from clinical research to the testing of advanced machine learning algorithms. Out of the 58 articles, six main categories are put in place and are detailed in Figure 2. These categories are obviously subjective and it is to be noted that some of the reviewed studies can have different purposes and thus belong to several categories.

The first main category gathers articles that intend to test a new activity classification method by developing an algorithm and/or a measurement device specific to their work [60,61]. Authors of these studies set up algorithms that allow, from previous annotated observations of participants performing activities (labeled training data), to determine which type of activity is performed during an analysis of a signal section (see Section 5 for more details on machine learning procedures for activity classification). Some of these studies point towards the study of machine learning systems [62,63,64], while others focus on other factors such as sensor implementation [65]. This is the group of aims that contains the greatest number of studies (28 papers).

Studies dedicated to clinical research use IMUs as a quantification tool for the study of specific cohorts [66,67,68] and constitute the second category (24 studies). Some of those clinical studies aim at quantifying patient’s pathology [6,7,27,69,70], while others quantify the therapeutic effect on the patient’s condition [5,68]. Some clinical trials focus on healthy participants, some compare the patients included with healthy participants [39,42,71,72], while others dwell specifically upon cohorts with a medical condition. Perriot et al. [7] intended to improve posture detection in COPD participants and Nguyen et al. [63] focused on activity classification in patients with PD. In such work, the aim is either to compare the results obtained on certain patients with specific pathologies to control patients or to evaluate the impact that changes in instrumentation (position of sensors, etc.) can have on the results observed in participants with pathologies.

The third group (18 studies) includes works focusing on the analysis of the different characteristics of implanted portable sensors (feasibility, placements, and comparisons between types of sensors or between sensors’ locations) [15,44,73,74,75,76]. Ellis et al. [17] compared the results of activity classification depending on the placement of the used sensors (hip or wrist) for instance.

The fourth group of works is dedicated to the evaluation of EE-related features (14 studies) [6,7,17,27,38,66,69]. Some of these studies intend to detect the amount of time spent in activities that require a greater or lesser expenditure of energy when carried out (sedentary activities for instance that is to say activities with a Metabolic Equivalent of Task (MET) is below 1.5. MET is the objective measure of the rate a participant expends energy depending on their mass).

Finally, papers comparing the conditions and results in free-living conditions with those obtained in controlled environments [40,72,74,77,78] and papers dealing with the detection of ancillary parameters (such as wear-time [79,80], walking bouts [72,75,81], strides or steps [40,71,81], etc.) constitute the fifth and sixth distinct groups of respectively 10 studies and five studies. Wear-time corresponds to the time a participant wears the sensors that he/she was provided with before the recording of his activities. Walking bouts are walking segments that are stationary.

## 3. Sensors

In this section, we will answer the first question raised by this review: What are the characteristics in terms of sensors configurations of the main methods for the study of PA in FLEs? Types, numbers, placements of sensors, their technical characteristics, as well as additional sensors’ details will be screened in this section.

### 3.1. Type of Sensors

**Figure 3:**
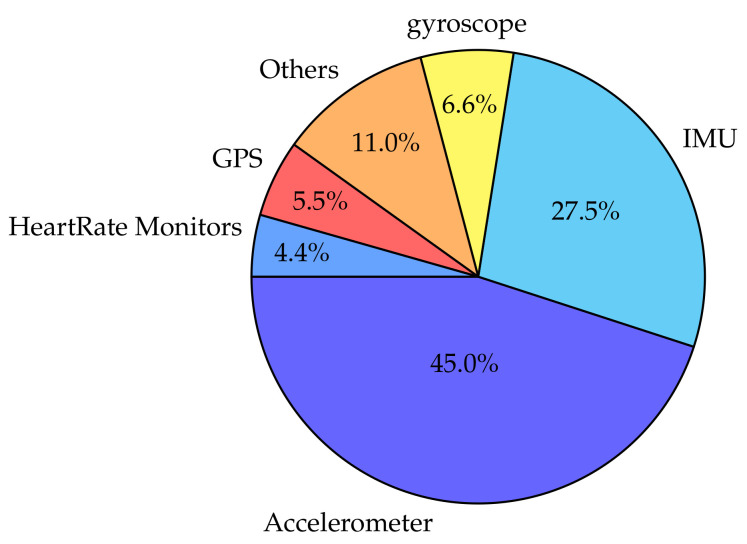
Proportions of the use of each kind of sensor in Free-Living Environments (FLEs) and Semi-Free-Living Environments (semi-FLEs).

All studies included in this review use accelerometers, gyroscope, IMUs [39,40,82], or an association of these three types of sensors. A referencing of the inertial sensors’ types among the 58 studies considered in the review is available in Figure 3. The devices’ brands for sensors that are used more than in one study are shown in Table 2.

It appears from Figure 3 that the two types of the inertial sensor most used for free-living applications are accelerometers (in particular the Actigraph accelerometers—see Table 2) and IMUs: 45% of accelerometers and 27.5% of IMUs. On the other hand, gyroscopes are rarely used individually (6.6% of the studies). Table 2 also shows that the devices used have storage and battery capacities that are quite similar and important compared to XSens, for example, which are often used for gait and locomotion analysis in controlled environments [26]. For the sake of comparison, the Shimmers, which are the most used IMUs in the reviewed studies, have 8 GB of internal storage and between 39 h and 69 h of battery autonomy, the Actigraph GT3Xs have an autonomy of 25 d and a 4 GB intern storage capacity, and the XSens (a popular sensor often used in controlled settings) have a maximum autonomy of 8 h and have no internal storage. Some recent studies use smartphones to retrieve details of the same parameters measured by the other types of motion sensors mentioned above. Three of these studies are included in this review [5,67,83].

### 3.2. Number of Sensors

**Figure 4:**
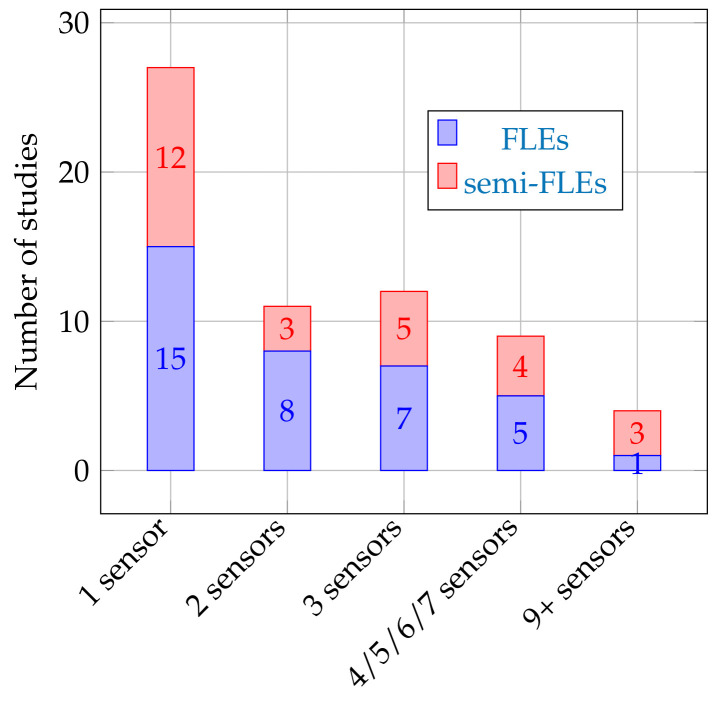
Distribution of studies according to the number of sensors associated with them: distinction between FLEs studies and semi-FLEs studies.

In several articles, protocols include the use of several sensors positioned on different locations on the body [40,44,62]. Figure 4 details the distribution of the studies according to the number of inertial sensors associated with them [17,38,62,66,73,78,80,84,85]. It shows that the majority of studies in FLEs or semi FLEs—even when using several IMUs, accelerometers, or gyroscopes—limit the number of the latter. Of all studies using more than one sensor, 67% use four or less sensors. [27,28,54,61,65,74,86]). Moreover, only three studies use more than ten IMUs—these studies use the IGS-180 suit consisting of seventeen IMUs [18,63,70]. Besides, 41% of the studies use only one sensor for all of their acquisitions [39,64,72,79]. This rather low number of sensors used in FLEs/semi-FLEs studies may appear surprising as the cost of inertial sensors has decreased and their dimensions have reduced in the last ten years (as detailed in Section 1). Yet, this could be explained by the fact that reducing the bulkiness due to inertial sensors is helpful in FLEs. With such a reduction, wearing sensors is less likely to serve as a reminder that a measurement is being taken. Thus, it avoids a potential appearance of Hawthorne syndromes [29]. This syndrome affects measurements in a clinical/controlled environment and reduces the spontaneous nature of certain movements observed during these measurements. In particular, although the figures do not allow for a definite conclusion, it seems that the higher the degree of freedom of the environment is, the lower the number of used sensors is. A reasonable explanation is that a too important bulkiness in complete FLEs paradigm is more complicated to manage (installation, charging of the sensors, etc.).

### 3.3. Sensors Placement

**Figure 5:**
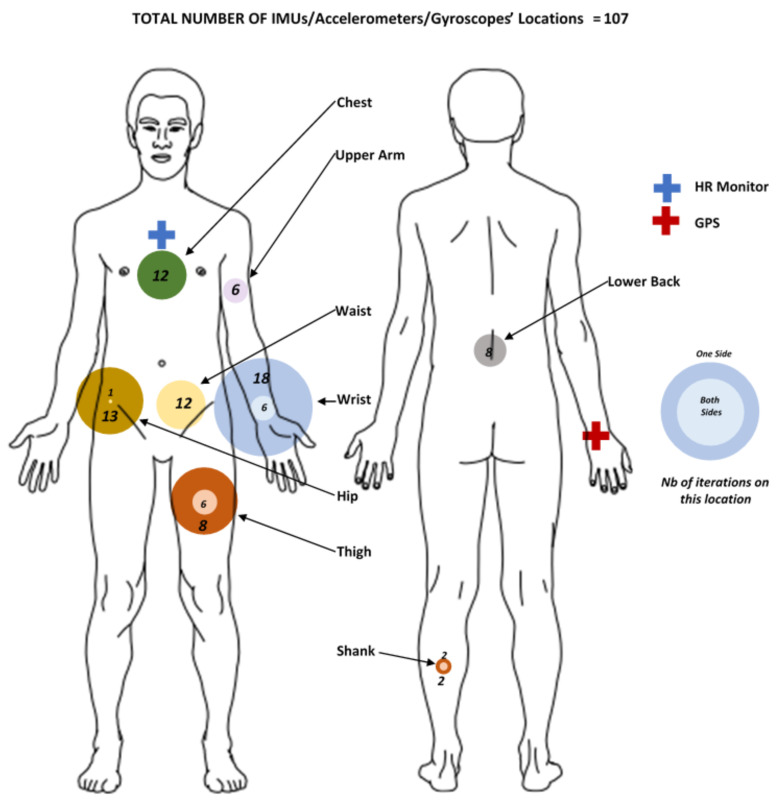
Sensors (IMUs, Accelerometers, gyroscopes, and other kind of sensors: GPS and Heart Rate Monitors) placements. Each circle displayed on the graph shows the proportion of use of the location associated with it (its radius is matched with the number of times that location is used in relation to the total number of locations used). Within these circles, another light-colored circle may be displayed which represents the proportion of the number of times the sensors placed on the location of the circle are placed on both sides of the participant. The remaining dark part represents the proportion of the number of times the sensors placed on the same location are placed on only one side of the participant. The numbers within each of the dark and light parts of the circles indicate the precise number of times each of these specific situations occur. The average position of the other sensors (GPS and HeartRate Monitors) are also indicated by crosses.

The locations of the sensors is also important as it conditions the types of results that can be obtained in a study. A mapping of the major placements for inertial sensors is available in Figure 5.

It appears that the sensor placements in FLEs are globally homogeneous, including both lower and upper body parts. A clear trend does not emerge even if the wrist position seems to be predominant (twenty-four percent of the studies use this location [64,69,79,83,87,88,89,90]). Several inertial sensors are sensors implanted on a watch (ActiWatch, GENEActiv, etc.), thus this high proportion of sensors placed on this location. In addition to this, it is reported in one study [73] that the participants tend to prefer this configuration since sensors are more comfortable and less cumbersome to wear on the wrist. However, it is important to notice that another study [91] has shown that 15.6% of their participants who wore accelerometers on the wrist violated the protocol for one or more days. Sensors were worn on the wrong hand during 6.9% of the days and during the periods of discrepancies, the daily PA was miscalculated by more than 20%. It therefore appear that behind the expected sensor locations, the correct placement of the device also has a significant effect on the results. It seems that very few studies conducted in open environments use portable sensors attached to the feet of participants. This can be explained by the fact that in unsupervised conditions, the use of a sensor on this location can present a detrimental congestion for the smooth running of the activities to be performed by the observed subject. These results clearly demonstrate the constraints and compromises that any protocol in FLEs relies on: getting the cleanest signals while still achieving a good acceptability.

### 3.4. Technical Characteristics

**Figure 6:**
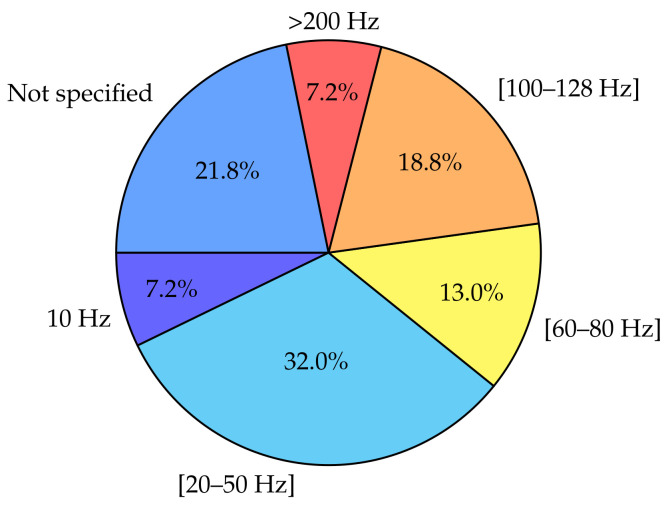
Distribution of sampling frequency among all articles. Note that three articles use sampling rates increasing from 10 Hz to 200 Hz in 10 Hz increments. They are thus counted in each corresponding slice of the pie chart.

As displayed in Table 2, sensors used in FLEs can be chosen according to several technical characteristics such as storage, battery life, or range of measurements. One of these important characteristics is the sampling frequency. This parameter influences both the level of precision of the processing and some practical considerations such as storage, size, or energy consumption. It is therefore important to locate and identify possible trends on the chosen value of this parameter according to the type of study and its associated objectives. The evaluation of daily PA by IMUs requires the selection of an adequate sampling frequency. The choice of this frequency must be based on the acceleration power of the human movement in order to be able to acquire all the data relating to it. In 1997, Bouten et al. [92] studied the acceleration power of human motion by distinguishing the upper body parts from the lower body parts. It was observed that the acceleration power in the upper body varies from 0.5 to 5 Hz. In the lower body, the heel strike can however produce acceleration frequencies up to 60 Hz. Knowing that, and depending on the sensors locations, sampling frequencies ranging from 10 Hz [74,93] to more than 200 Hz [88,94] have been used in the literature. As can be seen in Figure 6, there is large variety of sampling frequencies used articles. In several articles, the choice of the sampling frequencies is justified by considerations similar to those of Bouten et al. [92], and it is accepted in several publications that a sampling frequency greater than 20 Hz is an acceptable choice to capture most every day activities [16,60,83]. Indeed, according to Karantonis et al. [95], Bianchi et al. [96], and Khusainov et al. [97], all human body movements are within the range of 0 to 20 Hz, hence the importance of having sensors with sampling frequencies at least above 40 Hz (Nyquist criterion).

### 3.5. Additional Sensors

Although our review is not focused on non-inertial sensors, we can mention that several studies use inertial sensors associated with an additional sensor such as a GPS or a Heart Rate Monitor [6,43]. Some of them heavily rely on these sensors (GPS for instance) [41,43,68,98,99,100]. As far as GPS are concerned, their uses enable trajectory reconstruction [68,98,99,100] hence their usefulness in some cases (to correlate the data measured by the inertial sensors with the mapping of the movement of the participants in their environment). Tedesco et al. [48] review identifies certain types of GPS sensors that can be found in studies tracking activity but mixing up free environments (semi-FLEs and FLEs) with controlled environments. GPS tracking is sometimes used in studies in order to visualise the walking bouts or walking habits of participants. Nevertheless, it is necessary to remember that the accuracy of GPS is greatly degraded indoors, hence the almost unique use of these sensors in outdoor environments. Moreover, GPS do not provide valid data for vertical position. In some cases, GPS can also act as a good reference to correct the absolute positions of inertial sensors during horizontal movement phases. Indeed, they can be used to correct the drift errors of an algorithm named pedestrian dead-reckoning (with a Kalman smoother filter) intended to reconstruct the trajectory of participants and thus to be able to look for stationary walking phases [81].

### 3.6. Impact of the Sensors’ Set-Up

It should be noted that the variation that can be seen in the types of sensors used, their numbers, their placement on the participants, as well as their technical characteristics is quite marked. This variation may imply differences in signal retrievals, in their processing, and therefore in the accuracy of the activity classification calculation which is detailed in the Section 5. Indeed, several articles detail in particular the impact of different types of sensors by comparing, for example, different models of accelerometers used within the same protocols and on the same locations [44,74]. Comparisons can be made on the accuracy of the calculation of certain features or on the accuracy of activity classification. Furthermore, based on the analysis of these same types of results, some studies have highlighted the impact of differences in motion sensors’ placements on the retrieval and the use of data [17]. Besides, depending on the way the sensors are set up, different features can be computed. For instance, one acquisition performed with one sensor located on the lower back will not enable the same features’ retrieval than one acquisition with a sensor placed on the wrist. Researchers also have shown that sensor positioning errors could lead to variations (displacement within a body part due to insufficiently reinforced sensor mounting for instance). These variations can lead to a loss of orientation information that significantly affect the raw data [101]. Corrections (such as the use of orientation-robust features) to avoid these changes can be implemented when designing the measurement setup.

## 4. Protocols

In this section, we aim at providing insights on the different protocols used in the studies. Instructions to perform activities, activities’ and environments’ details, as well as inclusion criteria and annotations’ trends will be presented.

### 4.1. Instructions and Distinction between FLEs and Semi-FLEs Studies

As previously mentioned, one difficulty in the analysis of human movement is to achieve a balance in the experimental conditions. Indeed, although the goal is to record a movement as natural as possible, studies also aim at reaching the greatest possible accuracy. This forms a gradient of experimental condition more or less “natural” to which each experimenter will set the parameters. In the introduction in Section 1 of this review, we defined FLEs as environments that are not controlled by the experimenter. The participant thus has no indication of the environment and complete freedom of movement. On the opposite, we consider environments established by research teams as semi-FLEs. It includes laboratory, indoors or outdoors space, even replica of apartments. As will be seen in this section, it should be noted that these definitions are strictly based on the environment, but that there is also a gradient of freedom in the activities according to the instructions given.

#### 4.1.1. Instructions

**Figure 7:**
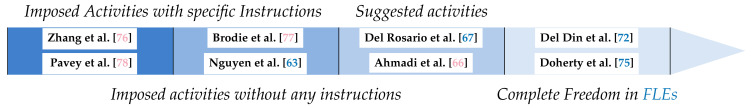
Frieze detailing the different types of instruction given to participants during FLEs and semi-FLEs studies. *Citations colored in red are citations of semi-FLEs studies. Citations colored in blue are citations of FLEs studies.*

Communicating the protocol to participants influences the way they perform the activities. The ideal protocol does not exist. For each protocol it is necessary to be consistent with the walking variable to be measured and the environmental conditions. Sustakoski et al. [102] observe through various protocols on the same participants a difference in walking speed according to the protocols. Overall, the paper prompts discussion on the notion that slight changes, such as walking on a computerize walkway or on the ground, can influence walking speed. Rehman et al. [103] compare the impact of walking protocols and gait assessment systems on patients with Parkinson’s disease, and underline the impact of the protocol on the activities performed. They observe at the level of two different walking protocols a difference in the participants’ performance (pace, rhythm, variability, and asymmetry). Thus, the format in which instructions are communicated is an important parameter in the protocol. In the literature, different types of instructions are observed, which leave more or less freedom of interpretation as we can see in Figure 7. There is a continuum of situations between semi-FLEs studies that still include precise instructions in the way of laboratory settings and FLEs studies where the subjects are completely free.

One type of instruction regularly found in semi-FLEs [5,16,60,71,76,78,82,85,89,104,105] is the presentation of the activities to be performed: how to perform them and for how long. These instructions are classified as “Imposed activities with specific instructions”. In this case, the participants have rigorous instructions and therefore no freedom of interpretation and completion of the activities. Other ways of instruction often found in semi-FLEs [66,67,88,106] are “Imposed activities without any instructions”. The participant is told which activities to perform but there is no precision on how to perform them. This protocol allows more freedom as to how perform the activities which makes them more natural. A third mean used in articles is the suggestion of activities. Indeed, some activities are suggested to the participants but without any additional instructions on how to perform them. In addition, the configuration of the environment itself can contribute to the suggestion (letting a pen on the floor which will imply that the participant leans forward to pick it up [18,40,43,63,77,93,99,107]). In this case, we are getting closer to a FLEs situation where the participant has complete freedom when performing activities. The last case observed mostly in FLEs situations is “complete freedom”. Participants are thus equipped with one or more IMUs and continue their daily and usual activities at home (including going to work, hobbies, and home activities) without any instruction or suggestion [6,7,17,27,38,39,44,60,69,72,73,74,75,80,89,91,100,104,104]. In the latter case, the participant is in the most natural conditions possible (both environment and activity). It is also noteworthy that a major aspect of the instructions are sometimes modified according to the studies: the exceptions in which the sensors must be removed (shower [80], sleep [100], etc.) according to the experts who set up the measurement protocols. This modification of the wear-time (which is, moreover, a parameter measured as precisely as possible by certain research teams) has a direct impact on the total recording time on a typical day. Participants from one study were even asked to remove their sensors whenever they felt skin irritation around the latter [65]. It should be noted that these requests for sensor removal may exist even when the instructions on the activities to be carried out are free: these two aspects are distinct.

#### 4.1.2. Environments

**Figure 8:**
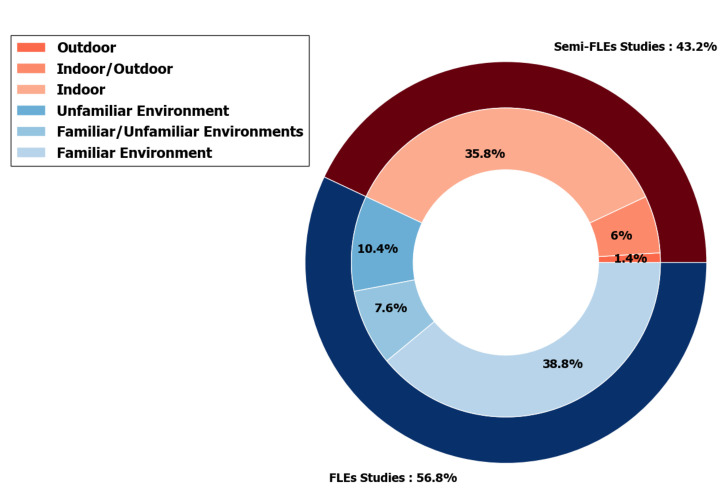
Number of studies focusing on Free-Living Environments (FLEs), semi-Free-Living Environments (Semi-FLEs), or both of them. The proportion of FLEs studies performed in Familiar, Unfamiliar, or both types of environments and the proportion of semi-FLEs studies performed in outdoor, indoor, or both types of locations are also detailed.

In addition to the instructions, the environments also play a part in the definition of FLEs/semi-FLEs conditions. The repartition of the studies included in this review according to these categories is displayed on Figure 8. It appears that the majority of the reviewed acquisitions are performed in FLEs (among the 67 reviewed acquisitions, 56.8% are in FLEs and 43.2% are in semi-FLEs). Acquisitions are specific recordings and several acquisitions can be put in place in one study. It may also be noticed that several studies include both FLEs and semi-FLEs acquisitions (15.5% of the reviewed studies).

Within FLEs studies, a distinction is made between certain papers depending on participants’ environment. Indeed, there are two types of environments: those that are familiar to the subject and those that are unfamiliar. Concerning articles using familiar environments, some studies in FLEs are limited to the habitats of the participants [6,17,27,37,38,41,69,72,73,79,80,83,84,91]. While others include all the environments frequented daily by the participant like library, gym, university, etc. [38,85].

As for semi-FLEs studies, they can be divided into two categories: indoor and outdoor studies. Indoor movements can be carried out on conveyor belts, on a 10 m path (or another defined distance) previously traced in a controlled environment (laboratory). Some studies are semi FLEs but take place in a reconstruction of an appartement: these environnements are simulated FLEs [18,43,70,106]. Others take place inside the laboratory, in infrastructures next to it, or both to be able to perform some activities: inside university campus [16,82] for instance. In those studies, participants are asked to perform a course that includes several activities that can not be performed only in a laboratory. Some studies imply indoor and outdoor parts [81].

**Figure 9:**
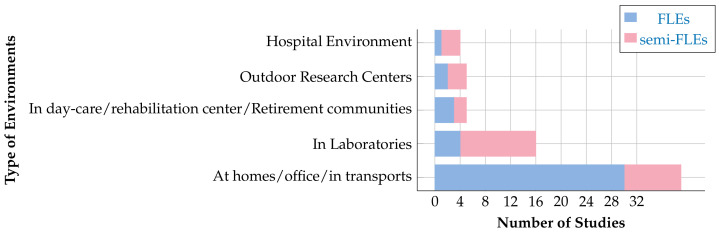
Details of the used Environments.

It is noteworthy that most FLEs studies include a controlled or semi-controlled environment section. It would seem that FLEs studies do not substitute for other experimental conditions, but provide access to other data that complement those obtained under semi-controlled conditions. The semi-FLEs FLEs and controlled conditions thus appear to be complementing each other to provide the most complete study of human activity. Figure 9 displays a detailed list of the environmental categories used in FLE and semi-FLEs studies. This figure also shows the number of times the measures are implemented in these types of environments. It can be seen that most of the protocols are implemented in environments familiar to the participants (homes, offices, etc.) but that some other arrangements are possible and quite frequently encountered (hospitals, rehabilitation centers, etc.).

### 4.2. Activities

**Figure 10:**
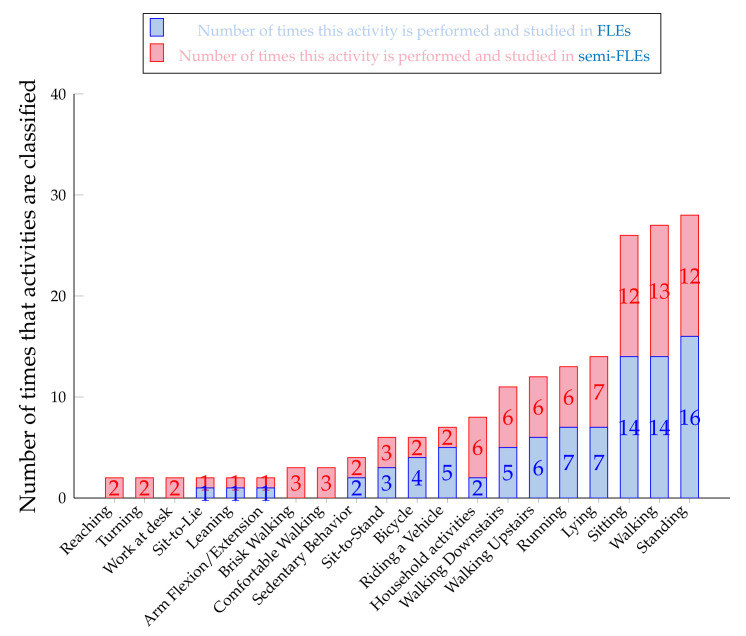
Details of the distribution of the studied activity types into FLEs and semi-FLEs. Total Number of Activities performed an studied in FLEs = 84. Total Number of Activities performed and studied in Semi-FLEs = 96.

In this section, the different activities used for the study of PA in free-living settings are presented. This section is organized in two parts: the first one being dedicated to full FLEs and the second one to semi-FLEs Figure 10 shows the distribution of the studied activities for both FLEs and semi-FLEs.

It appears that out of the eighty-two activities in FLEs, three activities are often analyzed: standing, sitting, and walking. Several articles simultaneously study these three activities [7,17,37,67,94]. The participants of these studies had to perform all of these three behaviors. A second slice of activities also seems to emerge and is composed of the following movements; running [17,78,93], lying down [54,62,67,94], going up and down a staircase [65,90]. It is notable that several studies are interested in the detection of postural transitions (sit-to-stand, stand-to-lie, etc.) [67,78].

Semi-FLEs enable a better control of performed activities and allow for more accurate annotations of these activities. It may result in an optimized computation of classifiers’ accuracies. As seen on Figure 10, the same initial conclusions as for the FLEs studies can be drawn here. The majority of the activities measured are standing, sitting, or walking, with a slight increase in the percentage of studies analyzing walking and running. Some new activities to be detected are also referenced in the semi-FLEs studies: turning and reaching [70]. semi-FLEs studies focused more on household activities [76,88] which are simpler to put in place and to annotate in such an environment. Besides, outdoor activities such as vehicle travels are less frequent (two semi-FLEs [93] vs. four FLEs [17,100]), probably because they are more difficult to set-up in this kind of environment.

It should be noted that some studies tried to detect groups of activities (sedentary activities [38,62,75], stationary activities [78,86,93], etc.) which included several specific activities (standing, sitting, lying down for the group of so-called stationary activities, etc.).

### 4.3. Measurements’ Durations

**Figure 11:**
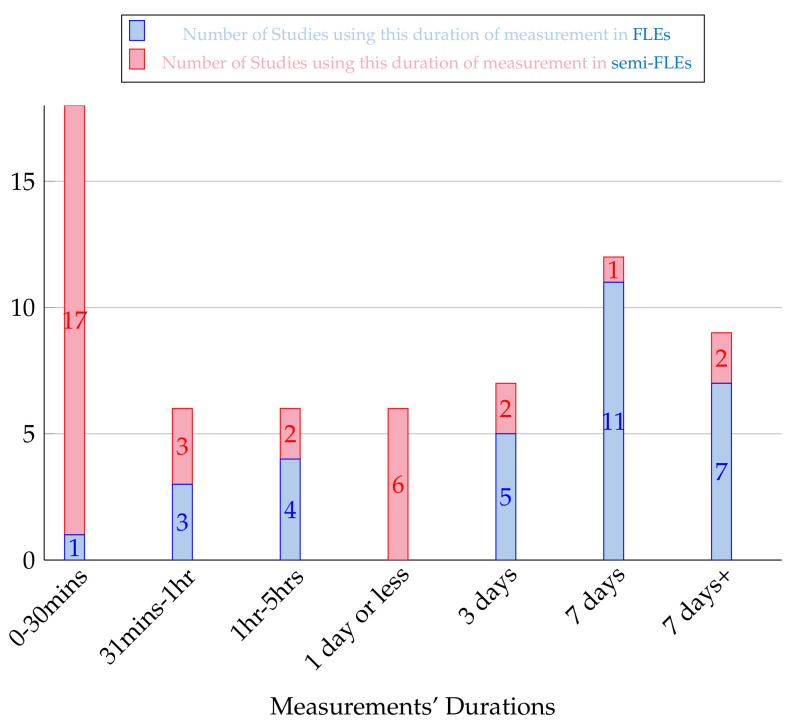
Measurements’ Durations in in FLEs and semi-FLEs studies.

Durations of recordings are also an important characteristic of the studies. Figure 11 displays the distribution of measurements’ durations for both FLEs and semi-FLEs studies. Strong differences between the two experimental conditions can clearly be observed.

FLEs studies mostly considered durations greater than 3 days. Indeed, of the 36 durations listed (one duration is not available in one article), 26% of the studies measure participants over seven days or more [17,65,84] and 73% percent of the studies measure subjects over three days or more [40,75,80]. This corresponds to the recurrent objectives in FLEs studies: to study extensive databases mimicking the phases of participants’ activities as closely as possible. These long studies allow to observe movements as spontaneous as possible, which is the interest of free-living studies. As confirmation of this point, it appears that there are only 8 articles that study the mobility of their subjects over less than one day.

It appears that the vast majority of registrations in semi-FLEs’ studies are completed in less than one day (85%). This is consistent with the fact that in semi-FLEs, deconstruction of motion phases is more easily feasible since the environment is controlled. A clear separation between the studies in semi-FLEs is observed around one parameter: the continuity or not in the measurements made. In fact, some research groups measure all movements by having them all performed in one single record [15,76,88,99], while other teams measure movements one after the other with a separation between each [42,77,106]. It is notable that there are more studies that evaluate their subjects continuously than non-continuously: 17 vs. 10. This corresponds to the desire of these studies to work on databases specific to the field of Free-Living (long and extensive). Besides, semi-FLEs studies tend to record their participants on short periods (17 semi-FLEs studies record less than 30 min). It adds up to the control such studies already have by constraining the space in which subjects perform activities. It should also be noted that there is a significant number of studies (five) that measure their participants over several days though [62,64].

### 4.4. Inclusion and Exclusion Criteria

**Figure 12:**
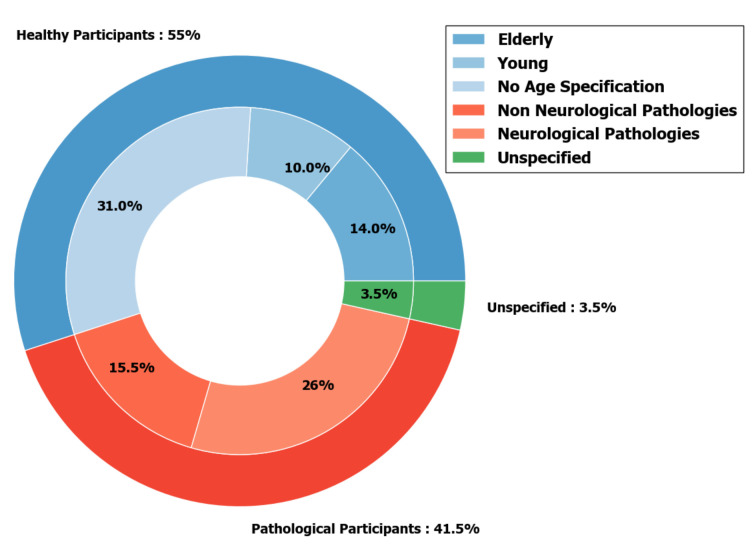
Distribution of studies according to participants state of health of participants. In shades of blue are represented the proportion of studies with non-ill participants (in light blue those with young participants, in intermediate blue those with elderly participants). The proportion of studies with ill participants is shown in shades of red. A distinction is made between neurological (dark red) and non-neurological (light red) diseases.

**Figure 13:**
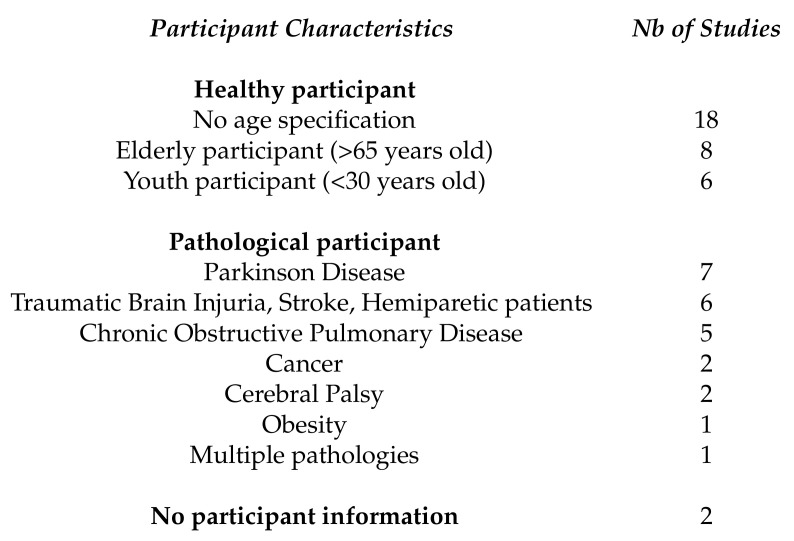
Detail of the number of studies according to the characteristics of included participants. Studies containing healthy participants are divided into three categories.

One of the advantages of ambulatories study is that they allow longitudinal follow-up of participants varying from a few hours to several days. It is particularly interesting for studying pathological patients without being limited to a one-off test of a few minutes during the hospital visit. This explains why a large proportion of pathological case studies are conducted in FLEs [6,7,17,27,28,39,69,79,84,89]. The definition of the inclusion and exclusion criteria determines the part of the population targeted by the study and therefore the precision of the study. Participants’ characteristics can sometimes affect their motor skills (taking medication that mimics the symptoms of another disease, co-morbidity of pathologies, etc.), hence the importance to describe the latter. Those characteristics (healthy or with specific diseases) are described in Figure 12 and further detailed in Figure 13.

In studies including only healthy participants (the majority of studies in this review) age is a recurring inclusion or exclusion criteria. Some studies focused on elderly populations, aged 65 and over [15,18,41,67,77,87,98,104]. Others focused on younger cohorts, with participants between adolescence and under 30 years of age [38,43,73,74,78,80,93,100]. The remaining studies used the age, gender, and BMI of the participants as a criteria [62,65,76,88,99], or only the average age of the included participants [16,54,60,108]. One study in particular took only the best survivors [85].

In the group of studies focusing on patients, a substantial part of studies focus on neurological pathologies like PD [5,63,70,71,72,82], cerebral palsy [40,66], accidental brain injuries like strokes [83,89,90,90,90], and traumatic brain injuries [39]. There is also a significant part of non-neurologic pathologies such as COPD [6,7,27,69] and obesity [17] where locomotion is also affected. All these pathologies have in common locomotion as biomarker which makes it a coherent choice.

For all the above-mentioned studies, a mandatory inclusion criterion is the official diagnosis of the disease under study [5,17,27,39,40,63,71,72,82,84,89,90]. Disease scaling questionnaires are sometimes necessary to include patients in the study like Parkinson pathology severity degree Hoehn and Yahr scale [5,70,71,72,82]. Functional tests can also be used as inclusion criteria as found in the studies with COPD participants for instance for whom a functional lung test is necessary as a diagnostic [6,7,27,28,69]. A recurring exclusion criterion is the co-morbidity of the studied pathology with another pathology that might have the same motor symptoms [39,72] and treatment that could also interfere with motor symptoms [72].

Finally, some inclusion and exclusion criteria are common to both study groups. Whether participants are pathological or not, general health information such as Body Mass Index are collected [40,43,76,99]. Furthermore, in all studies, physical (orthopedic or muscular) and cognitive impairments often are exclusion criteria [18,38,40,44,65,72] which are detected by questionnaires: Montreal Cognitive Assessment (MOCA) [70,72], Mini Mental State Exam (MMSE) [5,72], and the Geriatric Depression Scale (GDS) [41,72] for mental state. It is thus notable that the inclusion and exclusion criteria are grouped around age, health and BMI. These three characteristics allow a precise selection of participants and therefore of the study.

### 4.5. Annotations and Meta-Data

The analysis of the referenced articles shows that the “annotation of activities” aspect is essential in several studies, in particular to provide verification data and compute accuracy values for the classifiers used to classify activities. Sensors such as cameras, audio recorders, GPS, force plates, etc. can be used to annotate the timings of activities and compare them with those detected by the classifiers chosen by the investigators. The annotations are not only chosen to give an accuracy score for activity detection, but also sometimes to evaluate the number of Moderate Vigorous Physical Activities (MVPA): high energy level activities [73,74,75]. The annotations allow to evaluate the number of movements performed with a specific score of MET [27,28,38].

**Figure 14:**
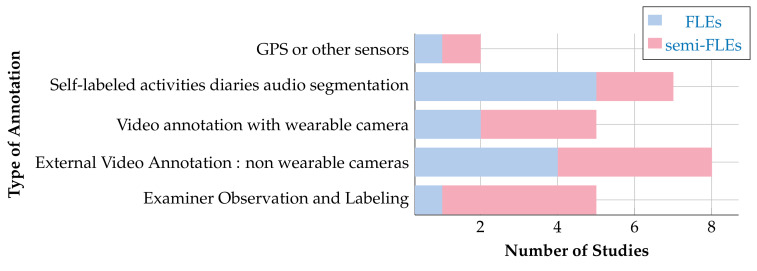
Distribution of the different kind of activities/physical behaviors/stationary phases annotations depending on environments.

As far as cameras are concerned, one study investigated how both inertial and vision sensors can simultaneously be used to enable human activity classification [109]. According to Figure 14, the most common devices for annotation are video cameras that have been widely used in FLEs [37,38,41,67,75,77,93,104] and semi-FLEs [16,82,106,110]).

Instead of additional sensors, some studies used direct manual annotations by experimenters [5,40,44,63,70,76,88,89,90,105,107]. Of course, those types of annotations are only relevant in studies that take place in laboratories (semi-FLEs). In FLEs, participants have to take notes into a diary that is given back to the experimenters at the end of the protocol or when the subjects take off the device [5,7,71,80,100,108]. In specific studies focused on the evaluation of EE, ground truth annotations can be provided by additional clinical exams. For instance, in [6,69] the analysis of urine samples is used to track the subject’s energy expenditure. The patient is asked to collect urine samples at a fixed time which is subsequently analyzed.

Observation of an examiner to annotate is more used in semi-FLEs than in FLEs where it is practically impossible to implement. Wearable or fixed cameras and GPS are used at a similar rate between FLEs and semi-FLEs studies. Besides, it appears that semi-FLEs enable a better control of performed activities and allow for more accurate annotations of these activities. It may result in an optimized computation of classifiers’ accuracies. It is interesting to note that GPS are sometimes used to detect stationary walking phases using trajectory reconstructions (using a standard inertial navigation algorithm termed pedestrian dead-reckoning (PDR) on a study [81]). This can allow to study the physical capacity of a subject on these phases and to potentially compare the results obtained in FLEs with those obtained in fully controlled environments.

Similarly, some questionnaires are also found to assess fatigue and ability to perform some physical activities and then study the physical capacity of a subject: Multidimensional Fatigue Inventory (MFI) [72], Nottingham Activity of Daily Living Scale [63], and Physical Activity Readiness Questionnaire (PAR-Q) [88,105]. These questionnaires are a means of completing the annotations because it permits to assess as good as possible the physical performance of patients.

## 5. Algorithms

At the exception of several studies such as [40,44,82], most studies (37 papers) rely at some point to classification algorithm that automatically detect the activities performed by the subjects [17,41,93]. Activity classification can be set up as a preamble to certain other objectives of the studies and makes it possible to achieve them. Such a classification is performed for instance to assess the physical activity of certain patients with diseases in a detailed manner [63]. It can also be needed to be able to compare efficiently laboratory acquisitions and FLEs’ acquisitions [77] and to compare the impact of the placement or of the types of wearable sensors [17,62]. It can also enable to validate the feasibility of a specific sensor to assess PA in free-living settings [38].

The common base of these studies, which is the classification of activities, allows for their comparison, particularly in terms of features, algorithms, and performance results. The aim of this section is therefore to focus on these studies and to provide an overview of the current state-of-the-art performances. Table 3 displays the main features retained in specific subcategories (described in Section 5.1), while Table 4 summarizes characteristics of these studies. Section 5.1 details the features that are computed from raw signals, and Section 5.2 provides insights on the classifiers used for activities’ classification.

### 5.1. Features

When portable sensors are installed on participants during their wandering in free-living conditions, research teams recovered raw signals whose nature differed according to the type of sensors implanted (linear accelerations for accelerometers, angular velocities for gyroscopes or both for IMUs, for example). From these raw data, it is possible to calculate a multitude of parameters that allow quantitative PA analysis. These parameters can be basic statistics (mean or standard deviation of accelerations over a certain duration for example) or derived from advanced algorithms (step lengths, average swing time, etc.). It has been shown in the clinical field that the latter can sometimes be used to define the quality of a walking category (rhythm, stability, springiness, etc.) of a participant [19].

Most simple features are Time-Domain (T-D) features that are only based on the timings of relevant gait events. T-D features are the parameters related to a notion of evolution in time of certain particularities of the obtained signal (standard deviation, means, etc.). More sophisticated features can be computed in the frequency domain, by focusing for examples on some relevant frequency bands: those are the Frequency-Domain (F-D) features. F-D analysis dwells upon the number of times some events occur in the recorded signals and to the notion of periodicity. From Table 4, it appears that the majority of the features retained for the classification of activities are T-D features (29 studies use T-D features [7,42,99,106] while only 15 studies use F-D features [17,41,77,78]). Table 3 details the most used parameters for each of these feature categories (T-D features, F-D features, and derived parameters) retained to set up the activity classifiers. T-D features such as variation coefficients, medians or standard deviation are calculated either on raw signals obtained by the sensors (linear acceleration and angular velocity) or on other specific parameters whose value is computed over each instant of time (vector magnitude and signal magnitude area). In addition, some specific parameters are used by several studies (Vector Magnitude (VM) [15,106], Signal Magnitude Area (SMA) [37,108] ...). VM incorporates the acceleration values from the cranio-caudal as well as the anterio-posterior and medio-lateral axes. SMA is a statistical measurement of the magnitude of a variable quantity (notably linear acceleration values). It it also to be noted that the sizes of the sample windows on which signals are analyzed are various (from 1 s [38] to 5 min [42]) but are often non-overlapping (only one study uses overlapping windows [67]).

### 5.2. Classifiers

When these parameters are calculated and listed, they can be used as variables for each observed activity in order to train classifiers. Every time an observation is done and associated with features’ values, a training example is created. A set of these training examples is created when several activities’ observations are performed. Each of this example is thus constituted of an input vector (features’ values of the associated observation) and of an output value (the type of activity performed during the associated observation) which is the label of the training example. The accumulation of these examples turns out to be labeled training data. Algorithms used in classifiers go through this data in order to determine the output value of an new incoming example. Some work can be put in place in order to define which features are either redundant (two features are redundant when their values are correlated) or irrelevant and which are thus to be kept to perform the classification: this is feature selection. Almost half of the studies classifying activities (18 of them) used feature selection processes such as Principal Component Analysis (PCA) [41,99] or others [54,83,93]) before setting up their classification system. The vast majority of these studies obtained classification accuracies (accuracy or sensitivity) beyond 85 percent. Accuracy is the proportion of true results (true negative or true positive) while sensitivity is the probability of a classification to identify a specific activity on a signal which truly corresponds to this activity. The classification algorithms are varied and each has its advantages and disadvantages according to the goals sought or according to the established databases. All algorithms referenced in Table 4 are supervised learning systems. This corresponds to a learning where the inputs, i.e., features, and the outputs, i.e., the associated types of activity, are known when data is processed. Concerning activity classification, unsupervised learning is much less predominant than supervised learning [111]. The fact that the labelling of activities can be carried out through annotations whose implementation is increasingly becoming more practical over the years (better quality cameras, use of trajectory reconstruction, etc.) may explain this difference.

It is important before beginning the discussion about classifiers and their associated precision rates to clarify that it is very complex, if not impossible, to compare the different studies selected on this specific topic. Indeed, depending on studies, classifications are carried out on a different number of activities carried out under conditions that sometimes differ drastically from one study to another (instructions, sensors, etc.). All these factors when added to others (positioning of sensors, cohorts, etc.) do not allow valid comparisons to be made between studies with regard to their classifiers and their associated algorithms.

It appears that the majority of the studies have an interesting average rate of classification precision (31 analyses obtain a precision or sensitivity higher than 85 percent [77,78,88,94]). It should also be noted that certain types of classifiers are recurrent, notably Random Forest (RF) (11 studies [83,85,100]) and Decision Trees (DT) (eight studies [38,61]), Support Vector Machine (SVM) (12 studies [43,76,84,94]), Bayesian approaches (Naive Bayes (NB)) (three studies [64,76,94]), K-Nearest Neighbor (KNN) (four studies [83,84,93,94]) and Neural Network (NN) (four studies [15,43,76,108] with notably Multi-Layer Perceptron (MLP)). Other classifiers (K-Means (K-M), Linear Regression (LR)) are also put in place in some studies but in less frequent manners and other methods and ancillary methods can also be implemented (Hidden Markov Model (HMM), Receiving Operating Characteristics (ROC), and Adaptation Detection Chain (ADC)). In addition, it is important to note that these papers may have several approaches. Indeed, some teams decided to compare or test several types of already known classifiers such as those cited above [67], while other teams combined some of these classifiers with other algorithms specific to their work in order to set up custom classification systems [38,88]. It is also possible that no existing classifiers are used and that everything is based on an algorithm designed for the study [62,63].

## 6. Discussion and Conclusions

In this article, we have reviewed 58 articles dealing with the use of IMUs in FLEs settings.

FLEs are environments that are not controlled by the experimenter. The participant thus has no indication of the environment and complete freedom of movement. On the opposite, we consider environments established by research teams as semi-FLEs; these can include the laboratory, indoors or outdoors space, and even replicas of apartments.

First, it appears from this overview that IMUs and accelerometers stand out as the most commonly used sensors. Their main location turns out to be the wrist as this is convenient to use it thanks to the development of watch sensors. The specifics of the free-living environments in which the studies take place is also a major factor of distinction between the reviewed works. It turns out that two types of such environments emerge: FLEs (environments that are not controlled by the experimenter) and semi-FLEs (environments established by research teams: laboratories, indoors out outdoors space, etc.). semi-FLEs’ conditions provide a smooth transition from controlled to full ambulatory settings that is to say FLEs’ conditions. Within those environments, the three most common types of activity studied in the reviewed works are walking, standing and sitting. According to the studies referenced in this review, the instructions given to participants to carry out these activities vary in their degree of explicitness (some impose activities, others suggest them, while others leave the subjects complete freedom in their natural environment). In addition, the recording times for taking measurements via motion sensors vary from one research team to another. The short recording durations (less than five hours) are often conducted in semi-FLEs’ studies while the FLEs’ studies setup longer recording times (three days or more). In all FLEs studies, annotation has a decisive role in data collection. The quantity, quality, and accuracy of the data thus depend on new ways of annotating, hence its diversity: diary, video, or tape recording. As soon as the implementation of sensors and the setup of the protocols are put in place and enable measurements, the computed data are to be processed. In several contexts, activity classification is a crucial step for the study of PA. The classification algorithms often rely on T-D features while F-D features are less commonly used. Additional features such as VM or SMA are also employed. After the selection of these features that a large part of the studies put in place (PCA, etc.), classifiers are used to classify the activities themselves. Certain types of classifiers are recurrent (RF, SVM, and DT) and constitute a popular choice for the task. Finally, it appears that the majority of studies in free-living conditions use protocols with a wide variety of implementations. Nevertheless, studies in FLEs conditions turn out not to be completely standard yet while semi-FLEs conditions are still commonly used. With regard to protocols’ characteristics such as instructions, measurement times or annotations, certain trends are emerging (in FLEs conditions: longer measurement times, annotations by the participants themselves or by monitoring systems other than examiners are becoming more popular, etc.). Within the studies evaluating the activity of participants in uncontrolled environments, some fairly clear tendencies seem to emerge: the massive use of accelerometers and IMUs compared to gyroscopes; the fact that these sensors are mainly positioned on the wrist, lower back, or waist; the predominance of activities such as walking, standing, and sitting in the protocols; and the use of time-domain features for the implementation of classifiers. These few observations, which make it possible to identify choices that are generally shared between studies concerning the implementation of devices and protocols in particular, may prove to be the main recommendations for future studies. Nevertheless, the differences between the studies are still notable and numerous, which prevents to bring out a standard approach to assess physical activity in free-living environments.

One recurring question in this review is linked to the notion of environment and to the distinction between semi-FLEs and FLEs. As a matter of fact, a shared feature of this continuum of protocols is the psychological dimension of the participants and its impact on their behavior. The white coat effect, for example, is caused by the presence of medical personnel or environment and influences the physiological measurements of the participants and their behavior [112]. Likewise, it is observed under experimental conditions that the presence of an experimenter influences the participant’s performance [113]. The Hawthorne effect, observed in people who are aware of participating in an experiment, which results in an increase in the motivation of the participants to perform the test and thus in the observed performance. Thus, the “artificial” side of a study is to be taken into account in the analysis of results, even in FLEs. Whether it is through social interaction or by knowing that we are part of a test, a psychological effect affects our behavior. Other psychological tendencies should be taken into account in the analysis of human physical activity. The trend to compare themselves to others illustrated by the “better than average” effect of not overestimating their abilities in comparison to others, it is a social comparisons effect that allow us to improve and measure our skills. There is also a current trend towards self-evaluation with the development of self-tracking applications and increasingly connected objects. Is the degree of accuracy of the IMUs identified in this review adequate for such use? Can the well-developed IMUs or other sensors reviewed in these studies be implemented in such conditions (self-evaluation) is it the field of more commercial sensors?

Another fundamental question is linked to the potential impact of this research field in public health. Indeed, the increase in NCDs, caused by the decrease in physical activity and the increase in the consumption of highly processed food with the rise of supermarkets and hypermarkets in the middle of the 20th century, is established. The collective awareness has led to the development of connected technology allowing the monitoring of energy expenditure and sports activity (smartwatch, pedometer in smartphone, connected weight watchers, etc.). Today, there is a willingness to control and measure physiological information, moreover: Would it be possible to integrate IMUs into connected objects in order to be able to quantify physical activities more precisely and in FLEs conditions? Would it be possible to link the use of IMUs to this wave of hyperconnection and to monitor one’s energy expenditure and physical activity? Thus the psychological dimension affects both decision making and actions. It is to be taken into account in the study of human activity, both from an experimental point of view, and for future monitoring in FLEs settings. Accurate sensors start though to become a must for FLEs physical activity assessment as participants perceived them more useful than smarthome settings for instance. IMUs are needed and appreciated. In particular, since the end of the 1990s, the smartphone has been continuously improved to become a technologically advanced object. It now includes features used in the biomedical and research fields (geolocation, magnetometer, accelerometer, high quality camera, etc.). It is a widely used devices, used both professionally [86,114] and personally. The smartphone is already used through the development of applications for medical purposes. There is a real need to reduce healthcare costs and the complexity of equipment in the biomedical field. However, the smart phone is relatively affordable, noninvasive biomedical facilities, which gives it definite advantages in the research community. Thus, the smartphone could prove to be an interesting device for studies in FLEs. Nevertheless, for some biomedical purposes, a high degree of accuracy is required. A recent study from 2020 [115] compares the use of smartphones and an application with inertial motion sensors used in research. Despite the good results in step detection, they observe a limitation in the use of the smartphone in the detection of physical activity in everyday life. Alongside the smartphone, other inertial sensors for consumer use are on the market (Fitbit) and allow activity detection. The comparison of these sensors with sensors used in the research community shows a good capability of consumer sensors for activity detection [116], but still less accurate than IMUs used in the research field which does not make it an adequate substitute for FLEs studies [117]. Furthermore, the lack of transparency and the difference in the algorithm used for step and activity detection with those sensors is a hindrance to the use of these sensors in the research community. Therefore, despite the advantageous qualities of the smartphone and other wide public sensors for studying in FLEs, it can be limited due to the lack of standardization and accuracy compare to IMUs used in research field. It should be noted that the distinction between sensors intended for the general public and sensors intended for the research field is subjective. It is based notably on the accuracy of the measurements and row data availability. Studies have evaluated the validity and reliability of consumer-grade sensors when used on elderly cohorts [118] and have shown that the performance of these sensors could be negatively impacted by the specificities of these populations and by certain conditions of use (outdoor use, walking aids, etc.) [119,120]. Thus, these sensors can hardly be used in a standard way in the research field as opposed to sensors considered as research-grade sensors.

Throughout this review, the diversity of the studies included is noteworthy. In spite of the search filters, the studies are very disparate, at protocols level, where we have shown the diversity of experimental conditions established. There is a splintering of studies according to the sensors brand used which implies a lack of the same sensors configuration (axis definition, accuracy, sampling frequency, etc.). Each brand has its own software and therefore of handling by scientists. This diversity is also found in the dataset used by each study. A large majority of studies record their own data with recruitment and testing of participants. This makes it possible to have customized data very specific to each study. Very few studies included in this review use already existing dataset [57,64,84], and even fewer aim at creating an open access dataset. There is no standardization of dataset between the different articles The diversity and lack of standardization of studies contributes to a compartmentalization of human activity research. This disparity may be a hindrance to research, which justifies the need for unification. One way to unify the studies would be the creation of a public and universally accessible dataset, as in the recent study by Garcia et al. [86]. Additional ways to unify the studies could be to work on a common goal such as the challenges (see, for example, in [121]), to have a shared aim for the whole community [121]. Although progress has been made in the detection of physical activity using inertial motion sensors, longer, larger, and more accurate studies are needed in order to be able to track patients longitudinally.

Finally, recent studies highlight the need for more accurate real-time activity classification, including the use of machine learning, in order to continue the study of activity in larger population samples. Several studies show that longitudinal monitoring in FLEs allows to follow the evolution of the patient’s [68] and therefore their condition. Activity classification was initially performed by pattern recognition. Although this method has permitted improvements in activity classification, it has limitations being sometimes heuristic and limited by human knowledge [122]. Since the last decade, machine learning, and more particularly Deep Learning, has allowed more accurate activity classification through NN. This enables to have a design feature and lean more higher level and meaningful features by training an end to end neural network. This is why deep learning is an ideal approach for activity classification and is being explored since the last decade. Deep Learning includes several models that permits different ways of data computation. Studies are now emerging with different models (Validation Neural Network, Convolutional Neural Network, Long Short-Term Memory (LTSM), etc.) and testing them on dataset in order to perform activity classification as accurately as possible. In particular, the use of recurring neural networks such as LSTM could be promising as they allow to remove the feature extraction step and directly work from raw signals. These techniques have successfully been used for activity classification in controlled environments [123]. The implementation of LSTM networks in FLEs or semi-FLEs could therefore represent a future possibility to improve the performances The use of IMUs sensors coupled with data analysis with Deep Learning models is becoming more and more frequent [124,125] and allows a more accurate analysis. This highlights, once again, the need for dataset pooling in order to have a larger library to allow better testing of these different models.

## Figures and Tables

**Table 1:** Comparison of our review to other existing reviews dealing with the use of wearable motion sensors in free-living settings. 1 → De Bruin et al. [46] (2008), 2 → Murphy [49] (2009), 3 → Yang and Hsu [52] (2010), 4 → Schwickert et al. [55] (2013), 5 → Gorman et al. [45] (2014), 6 → Attal et al. [53] (2015), 7 → Byrom and Rowe [47] (2016), 8 → Tedesco et al. [48] (2008), 9 → Tedesco et al. [48], 10 → Wang et al. [57] (2017), 11 → Narayanan et al. [54] (2019), 12 → Henriksen et al. [56] (2020), 13 → Frechette et al. [51] (2019),14 → de Oliveira Gondim et al. [50] (2020), 15 → This Review.

		1	2	3	4	5	6	7	8	9	10	11	12	13	14	15
Specific Factors	Focused on FLE/Semi-FLE	*√*				*√*		*√*		*√*						*√*
	Considering all cohorts	Elders	*√*	*√*	*√*	*√*	*√*	COPD	Elders	*√*	Elders	*√*	*√*	MS	PD	*√*
Sensors	Types of Sensors	*√*	*√*	*√*	*√*	*√*	*√*	*√*	*√*	*√*	*√*	*√*	*√*	*√*	*√*	*√*
	Number of Sensors			*√*	*√*			*√*	*√*			*√*			*√*	*√*
	Placements	*√*	*√*	*√*	*√*	*√*	*√*	*√*	*√*	*√*	*√*	*√*		*√*	*√*	*√*
	Technical Characteristics			*√*	*√*							*√*			*√*	*√*
	Additional Sensors								*√*		*√*					*√*
Protocols	Instructions’ Details							*√*								*√*
	Measurement’s Durations		*√*					*√*				*√*	*√*			*√*
	Annotations								*√*			*√*		*√*		*√*
Algorithms	Features			*√*			*√*		*√*	*√*	*√*	*√*		*√*		*√*
	Classifiers						*√*		*√*		*√*	*√*				*√*

**Table 2:** Details of used inertial sensors. dps: degrees per second.

Device Names	Type of Sensors	Battery Life	Storage	Nb of Studies	Accelerometer Range	gyroscope Range	Sampling Frequency
Actigraph (*: GT3X)	Accelerometers	25 days	4 GB	14	±8 g	NC	30–100 HZ
Shimmer (*: Shimmer3)	IMUs	39–69 hrs	8 GB	7	±2 g (to ±16 g)	±250 dps (to ±2000 dps)	512 Hz
ActivPal (*)	Accelerometers	10+ Days	NC	5	±2 g	NC	20 Hz
Physilog GaitUp (*Physilog 4)	IMUs	23 hrs	8 GB	3	±2 g (to ±16 g)	±250 dps (to ±2000 dps)	1–500 Hz
AX3	Accelerometers	14 days at 100 Hz	512 MB	3	±2 g (to ±16 g)	NC	12.5–3000 Hz
GENEActiv	Accelerometers	30 Days	0.5 GB	3	±8 g	NC	10–100 Hz
IGS-180 Suit (Xsens)	IMUs	6 hrs	None	3	±16 g	±2000 dps	100 Hz
Dynaport (* MoveMonitor)	Accelerometers	14 Days	1 GB	2	±2 g (to ±8 g)	NC	50–200 Hz
Sensewear	Accelerometers	14 days	20 days	2	±2 g	NC	50–60 Hz
Hookie AM20	Accelerometers	NC	NC	2	±16 g	NC	100 Hz
Actiwatch (Actiwatch 2)	Accelerometers	30 Days	1 MB	2	±0.5 – ±2 g	NC	32 Hz
Actical	Accelerometers	194 days	32 MB	2	±0.025 – ±2 g	NC	32 Hz
Empatica E4	Accelerometers	1–2 Days	60+ hrs	2	±2 g (to ±8 g)	NC	32 Hz

**Table 3:** Details of the features mostly selected to feed activity classifiers according to their associated categories (T-D Features, F-D Features, and derived parameters).

T-D Features	F-D Features	Derived Parameters
Mean Coefficient of Variation Standard Deviation Variance Min, Max Median Autcorrelation Coefficients 25th and 75th percentile	Dominant Frequency Dominant Frequency Magnitude Spectral Power Spectral Energy	Speed Step/Stride Time Step/Stride Velocity

**Table 4:** Summary of all the specificities of the studies selected for this review which classify performed activities. The features retained as well as the nature of the classifiers and their precision are detailed (acc: accuracy, se: sensitivity).

				Features								Algorithms	
Date	Reference	Number of Activities	Time Resolution	Features Selection	T-D Features	F-D Features	VM	SMA	Angles, ROM	Corr	MAD	Classifier(s) Type	Accuracy
2010	Khan et al. [108]	15	3.2 s		*√*	*√*		*√*	*√*			NN	Acc = 97.9%
2012	Clements et al. [99]	2	4 s	PCA	*√*	*√*						Other	Acc = 90% → 95%
	Tran et al. [83]	NC	NC	Performed								K-M, KNN, RF SVM, RDF SVM	Acc = 71% → 96%
	Zhang et al. [76]	15	NC			*√*						DT, SVM, NB, LR, NN	Semax = 97.20% avg
2013	Lockhart et al. [16]	NC	NC									Wavelet	Se = 96.78%
	Leutheuser et al. [88]	13	NC		*√*	*√*						KNN/SVM	Acc = 89.6% avg
	Perriot et al. [7]	3	5 s		*√*				*√*			Other	Se = 77% → 94%
2014	Gao et al. [94]	8	1 s	Performed	*√*	*√*		*√*	*√*	*√*		SVM, NB, kNN, DT	89.5% → Acc=96.8%
	Del Rosario et al. [67]	8	1.25 s									DT	Se = 69.2% → Acc = 82%
2015	Chernbumroong et al. [15]	13	NC	FC	*√*	*√*	*√*					MLP, SVM, RBF, NN	Acc = 97.20% avg
	Massé et al. [106]	8	NC									Other	Acc = 90.40%
	Papadopoulos et al. [87]	NC	NC	NC								Other + ADC	
2016	Ayachi et al. [18]	8	160 ms	FC	*√*				*√*	*√*		ADC	Se = 97% avg
	Hu et al. [100]	4 Groups	30 s	RF	*√*							RF (100 DT)	Acc = 99.71% avg, Se = 84.62% → 99.9%
	Brodie et al. [77]	1	1.2 s	Performed	*√*	*√*						DT	Acc>97%
	Ellis et al. [17]	4	1 min	RF	*√*	*√*	*√*		*√*	*√*		RF, HMM	Acc = 84.6% → 89.4%
	Kerr et al. [85]	5	1 min		*√*		*√*					RF	Acc = 51% → 77%
	Alam et al. [41]	13	NC	PCA	*√*	*√*						RF	Acc = 91.80%
	Weiss et al. [42]	3	5 min		*√*	*√*						LR	Acc = 90.2% → 94.3%
2017	Pavey et al. [78]	4 Groups	10 s	Performed	*√*	*√*						RF	Acc = 92.70%
	Nguyen et al. [63]	7	NC		*√*				*√*			Other	Acc = 100% avg Se = 97.6%
	Fullerton et al. [93]	8	3 s	Performed	*√*	*√*	*√*	*√*				DT, SVM, kNN	Acc>95%
	Chowdhury et al. [64]	5	5 s	Correlation Based						*√*		NB	Acc = 65% → 79%
2018	Nguyen et al. [70]	7	NC		*√*				*√*			Other	Acc = 90% avg, Se = 90.8%
	Vähä-Ypyä et al. [62] (FLE)	4	6 s		*√*				*√*		*√*	ROC Curve	Other
	Vähä-Ypyä et al. [62] (Semi-FLE)	4	6 s		*√*				*√*		*√*	ROC Curve	Other
	Derungs et al. [89]	51	1 min		*√*							LR	Se = 80%
	Nazarahari and Rouhani [60] (FLE)	12	NC			*√*			*√*			SVM	Acc = 99%
	Kerr et al. [84]	6	5 s		*√*		*√*					SVM, NB, DT, RF, HMM, kNN	Acc > 80% (RF)
	Fiorini et al. [43]	8	3.5 s		*√*						*√*	DT, SVM, NN	Acc > 92%
	Cajamarca et al. [61]	8	NC		NC	NC						DT	Acc = 93.50%
	Ahmadi et al. [66]	4 Groups + Others	10 s	RF	*√*	*√*						RF, SVM, BDT	Acc = 76.1% → 89%
2019	Awais et al. [37]	1	1 s to 10 s	Three Methods	*√*	*√*	*√*	*√*				SVM	Acc>80%
	Narayanan et al. [54]	12	5 s	Performed	*√*	*√*	*√*		*√*	*√*		RF	Acc = 53% → 99%
	Crowley et al. [65]	8	2 s		*√*							Custom	Se = 63% → 100%
2020	Marcotte et al. [38]	1	1 s		*√*		*√*					RF, DT	Se = 60.4% → 93.5%
	Garcia-Gonzalez et al. [86]	4	1 s	Performed	*√*							SVM	Acc = 67.22% avg
